# Malignant melanoma and papillary thyroid carcinoma that were diagnosed concurrently and treated simultaneously: A case report

**DOI:** 10.3892/ol.2014.2642

**Published:** 2014-10-27

**Authors:** ALPASLAN OZGUN, TOLGA TUNCEL, LEVENT EMIRZEOGLU, SERKAN CELIK, OGUZ BILGI, ABDULLAH HAHOLU, MUAMMER URHAN, BULENT KARAGOZ

**Affiliations:** 1 Department of Medical Oncology, Gulhane Military Medical Academy Haydarpasa Training Hospital, Istanbul, Turkey; 2 Department of Pathology, Gulhane Military Medical Academy Haydarpasa Training Hospital, Istanbul, Turkey; 3 Department of Nuclear Medicine, Gulhane Military Medical Academy Haydarpasa Training Hospital, Istanbul, Turkey

**Keywords:** malignant melanoma, papillary thyroid carcinoma

## Abstract

Malignant melanoma can be successfully treated when it is identified in its early stages, but the disease is associated with a poor prognosis when it is detected in an advanced stage. Papillary thyroid carcinoma is a thyroid cancer that has a good prognosis. The present study reports a rare case of malignant melanoma and papillary thyroid carcinoma that were diagnosed concurrently and treated simultaneously. The present patient was a 37-year-old male, in whom examination of a skin biopsy that was obtained from a lesion in the right retroauricular region revealed the lesion to be consistent with malignant melanoma. The patient underwent radical neck dissection upon the detection of malignant melanoma metastasis to the sentinel lymph node. Metastases of papillary thyroid carcinoma were detected in four out of 38 lymph nodes. The patient was then diagnosed with papillary thyroid carcinoma and underwent total thyroidectomy. The patient was administered with high-dose followed by moderate-dose interferon-α therapy for the treatment of malignant melanoma. The patient also received concurrent radioactive iodine therapy for the treatment of papillary thyroid carcinoma, at the same time as the interferon therapy. The two primary tumors of the patient were treated successfully. During therapy, no serious side-effects were observed, with the exception of fever caused by high-dose interferon therapy. Malignant melanoma and papillary thyroid carcinoma may occur concurrently, although this is rarely observed. The present study reports a rare case that demonstrates that the two tumors can be successfully treated simultaneously.

## Introduction

Malignant melanoma is a skin cancer that is caused by the malignant transformation of melanocytes. The disease rarely occurs in the mucosal tissues and uvea. Malignant melanoma can be successfully treated if it is detected in the early stages of development. Surgery is the standard treatment for early stage melanoma. In addition, interferon-α has been approved for adjuvant treatment following excision in disease-free patients at high risk of recurrence. However, the prognosis associated with advanced-stage malignant melanoma is poor; the disease accounts for ~4% of all skin cancers, but results in 80% of skin cancer-associated mortality (1,2). In total, 75% of thyroid cancers are papilliary thyroid cancer, which is associated with a good prognosis (3). Papillary thyroid cancer may result from exposure to radiation. Surgery is the standard treatment for papillary thyroid cancer. Approximately four-six weeks following surgical removal of the thyroid, radioiodine therapy may be administered to detect and remove any metastasis and residual tumor tissue in the thyroid. Second primary tumors may develop in patients with malignant melanoma. Previous studies have reported associations between malignant melanoma and a wide variety of malignancies including second primary melanoma, non-melanoma skin cancer, central nervous system tumors, Hodgkin’s lymphoma, non-Hodgkin’s lymphoma, leukemia, breast carcinoma, ovarian carcinoma and papillary thyroid carcinoma (4). However, papillary thyroid carcinoma rarely co-occurs with malignant melanoma. The present study reports the case of a rare co-occurrence of malignant melanoma and papillary thyroid carcinoma, which were treated simultaneously. Written informed consent was obtained from the patient.

## Case report

### Diagnosis

**Figure 1:**
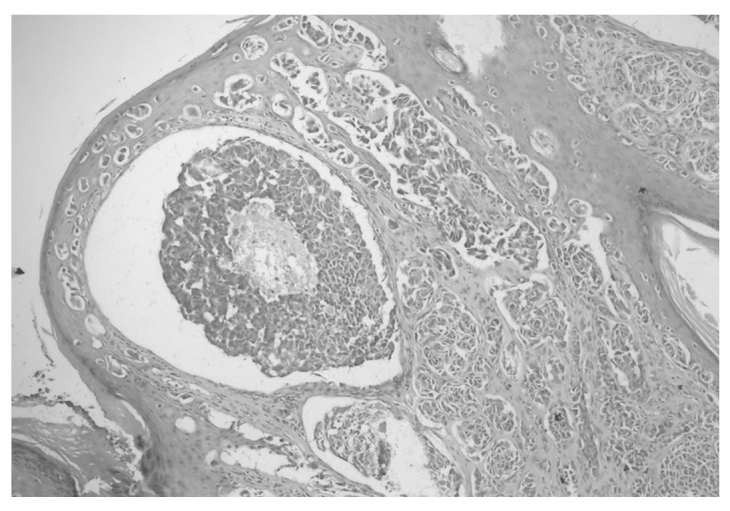
Light microscopy image of malignant melanoma (hematoxylin and eosin stain; magnification, ×100).

A 37-year-old male patient presented to the Gulhane Military Medical Academy Haydarpasa Training Hospital (Istanbul, Turkey) with a one-year history of a growing nevus located in the right retroauricular region that had been undergoing changes in color. An excisional biopsy was performed on the suspicious skin lesion. Pathological examination suggested that the lesion was a nodular-type malignant melanoma, Clark level IV and with a Breslow thickness of 3 mm (Fig. 1). Neck ultrasonography revealed two lymphadenopathies in the right jugular chain, the largest measuring 11 mm. Thoracic and abdominal computed tomography and bone scintigraphy did not reveal the presence of metastasis. Lymphoscintigraphy of the right neck was performed using Tc99m. The procedure revealed uptake in two foci located in the right retroauricular region and the right upper cervical lymph nodes. The patient then underwent extensive re-excision and sentinel lymph node biopsy. Metastasis of the malignant melanoma was detected in one of the sentinel lymph nodes.

### Treatment

**Figure 2:**
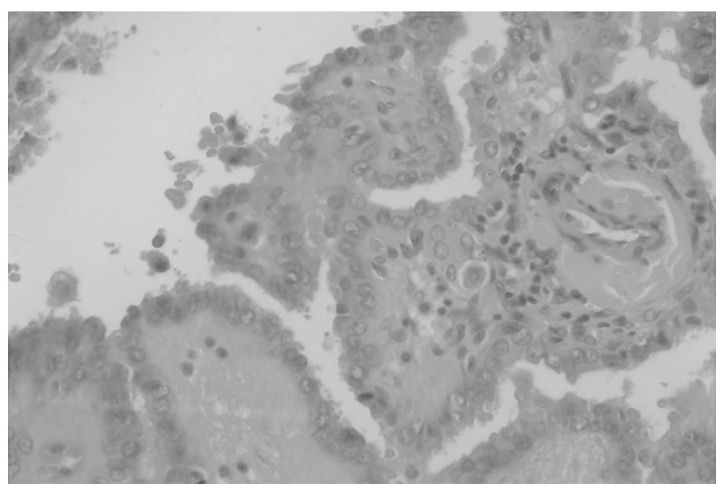
Light microscopy image of papillary thyroid carcinoma (hematoxylin and eosin stain; magnification, ×200).

The patient subsequently underwent modified radical neck dissection and pathological examination revealed the presence of papilliary thyroid carcinoma metastasis in four out of 38 lymph nodes and also revealed reactive hyperplasia in the remaining 34 lymph nodes. The patient subsequently underwent total thyroidectomy and the pathological examination revealed a classical variant of papillary thyroid carcinoma (Fig. 2). Imaging examinations did not reveal distant organ metastasis associated with either primary tumor. The patient was initially intravenously administered with high-dose interferon therapy (20 MU/m^2^) five days a week, for four weeks, to treat the malignant melanoma. In order to treat the papillary thyroid carcinoma, the patient was administered with low-dose (5 mci) radioactive iodine therapy in the second week of the high-dose interferon therapy. The high-dose interferon therapy was followed by moderate-dose interferon therapy (10 MU/m^2^), which was intravenously administered three days a week for 48 weeks. The patient was also administered with high-dose radioactive iodine (150 mci) therapy in the eighth week of the moderate-dose interferon therapy.

The two primary tumors observed in the present patient were successfully treated. No serious side-effects were observed during therapy, with the exception of a fever caused by the high-dose interferon therapy. The patient continues to be followed up by the Gulhane Military Medical Academy Haydarpasa Training Hospital and is disease-free at present. Moderate-dose interferon therapy and thyroid hormone replacement therapy continue to be administered.

## Discussion

Advanced-stage malignant melanoma is associated with a poor prognosis. However, the therapeutic agents identified in previous years have provided significant improvements in patient survival (2). Papillary thyroid carcinoma is the most common type of thyroid cancer, and is associated with a good prognosis (3). Second primary tumors may occur in patients with malignant melanoma. In a study by Bhatia *et al*, 37 out of 585 patients (6.3%) with malignant melanoma were found to possess a second primary tumor. Of these patients,23 had other skin cancers and the remaining patients had lymphoma and breast, bladder, lung, prostate and cervical cancer lesions (4).

Papillary thyroid carcinoma rarely occurs as a second primary tumor in patients with malignant melanoma. Kim *et al* reported a case of malignant melanoma in which concurrent papillary thyroid carcinoma manifested with hypothyroidism (5). Goggins *et al* reviewed 73,274 patients with malignant melanoma who were diagnosed between 1973 and 2000, and found that there was a 2.17-fold increase in the risk of developing thyroid cancer as the concurrent second primary tumor (6). Previous studies have reported a higher frequency of BRAF mutation in patients with malignant melanoma and thyroid cancer compared with patients suffering from other types of cancer (7–10). In the study by Goggins *et al*, a high frequency of BRAF mutation in the patients with malignant melanoma and thyroid cancer was suggested to be the cause of the co-occurrence of the two diseases (6).

In the present study, a radical neck dissection was performed following a sentinel lymph node biopsy that detected the presence of metastases from malignant melanoma, which also resulted in the detection of metastasis from papillary thyroid carcinoma in the four excised lymph nodes. Therefore, the present patient was diagnosed with papillary thyroid carcinoma as a second primary tumor and the two primary tumors were successfully treated.

It should be considered that patients with malignant melanoma may also possess concurrent papillary thyroid carcinoma as the second primary tumor, and the lymph nodes removed during radical neck dissection must be carefully examined from this perspective. Furthermore, it should be remembered that the two tumors can be treated simultaneously and that the treatment of either tumor should not be delayed.
